# Secondary Trauma: Emotional Safety in Sensitive Research

**DOI:** 10.1007/s10805-019-09348-y

**Published:** 2020-01-07

**Authors:** Emma Williamson, Alison Gregory, Hilary Abrahams, Nadia Aghtaie, Sarah-Jane Walker, Marianne Hester

**Affiliations:** School for Policy Studies, University of Bristol, 8 Priory Road, Bristol, BS8 1TZ UK

**Keywords:** Sensitive research, Gender based violence, Vicarious trauma, Secondary trauma, Researcher safety

## Introduction

Secondary trauma (ST) refers to the impact of indirect exposure to traumatic experiences; effects which can be ‘disruptive and painful’ and can ‘persist for months or years’ (McCann and Pearlman 1990). The effects, as described by McCann, in relation to working directly with clients, are considered to be a usual response which results from witnessing a distressing traumatic event or from knowledge about such an event, particularly if the person is connected with the victim-survivor (Figley 1998). ST is one of a number of terms used somewhat interchangeably (including vicarious trauma, burnout, compassion fatigue) to convey ideas about the transference, or rippling-out effects, of trauma from the original incident and the original victim-survivor. Burnout is more usually related to the demands of work (including caregiving and studying) and its contextual components, such as long hours, insufficient support or control, and heavy workload, than the specific nature of work involved, and thus may be different, if overlapping, with the topic we are looking at (Freudenberger 1974). Brown (2017) refers to the Maslach Burnout Inventory (2015), which differentiates burnout from other forms of exhaustion or depression, due to its inclusion of an element of compassion fatigue. It is this aspect which those with caring responsibilities find particularly difficult to acknowledge and address. Because this previous research has often focused on front line workers rather than researchers and we know collectively very little about the impacts of studying trauma on this group, we use the term ST within this paper as a more descriptive term which doesn’t imply an outcome or particular impacts, yet can include a similar range of impacts found in the phenomena of burnout or compassion fatigue. The term secondary trauma is not ideal but it is important to recognise where this paper sits in relation to the wider literature on the impacts of working with trauma as the focus of ones work. We also use the term ‘emotional safety’ to recognise ways in which the potential for ST can be acknowledged.

The American Counselling Association describes ST as the ‘emotional residue of exposure’, explaining that it results from people witnessing trauma (by direct exposure or by hearing narratives about it), and thus becoming ‘witnesses to the pain, fear, and terror that trauma survivors have endured’ (American Counselling Association 2010). The idea that front-line emergency responders, and people working therapeutically with those who have experienced trauma, might experience vicarious impacts is not new (Maslach 2015). Professionals considered to be at potential risk of traumatisation have historically included: rescue workers, police officers, military personnel, emergency healthcare staff, and counsellors/therapists (Brown (2017); McCann and Pearlman 1990; Ursano et al. 1999). Increasingly, it has become recognised that there are many more groups of people who might experience ST, particularly those in a range of ‘helping professions’, who may assist trauma survivors, including: humanitarian workers, social workers, suicide helpline workers, a wide range of healthcare professionals, justice system professionals, journalists, and faith leaders (Rafferty 2004; Figley 1998; Day et al. 2006; Pryce et al. 2007; Shah et al. 2007; Sansbury et al. 2015).

So, where do researchers fit in this picture? We intuitively understand that front-line professionals exposed to the traumatic and sometimes horrific experiences of others might be affected, but the shift towards acknowledging the impacts on researchers working with information about traumatic events or with traumatised individuals has been slower (Dickson-Swift et al. 2008, 2009). Is that because researchers, on the whole, do not experience these affects? Or because it is not considered or acknowledged? Or is it because, as considered below, this type of impact can be cumulative?

In the past, we might have considered researchers outside of the ‘at-risk’ groups for ST for a number of reasons: they rarely see the traumatic events that people experience, they rarely interact with people who have experienced trauma for more than a handful of occasions, and they do not have an explicit helping role in the situation. In addition, part of the reticence about recognising the potential for researcher ST may be, in part, due to traditional views of academic scientific endeavour as objective, detached and neutral, where researchers are not supposed to *feel* anything (other than perhaps satisfied or frustrated) about the work they undertake. In reality, research is rarely an entirely neutral process, and researchers are often neither impassive nor unaffected by the research they conduct (Hallowell et al. 2005). This is particularly true for research using qualitative methods, where people may narrate their experiences in depth, though we would argue that it can also be the case for studies using a quantitative paradigm.

The topic of the research is, perhaps, most crucial in terms of risk of ST. This paper focuses on the field of Gender Based Violence (GBV). The World Health Organisation (WHO) identifies the most common risk for fieldworkers in this area as the ‘emotional toll of listening to women’s^1^ repeated stories of despair, physical pain and degradation’ (Ellsberg and Heise 2005), with interviewers describing the imprint that bearing witness to violent narratives had had on them:

*When I heard stories about women [sic] being beaten and tied up, I would leave there feeling desperate… I would be a wreck, and my supervisor would tell me “get a hold of yourself, you cry for every little thing.” But how could I control myself? I couldn’t stand it… I would try, but sometimes it was impossible, and I would burst into tears during the next interview…* (Ellsberg and Heise 2005: 42).

The practical guide produced by the WHO for researchers of GBV clearly describes the ethical responsibilities within research; not only to keep all parties physically safe, and to minimise participant distress, but also to consider the impact that this work has on researchers’ wellbeing (Ellsberg and Heise 2005). Of course, these are not unique considerations for those researching GBV, researchers working with trauma in other fields may encounter similar. In fact, a brief scoping of the literature indicates that secondary distress has been raised as an issue in recent years by researchers working in a number of fields, including suicide (McKenzie et al. 2016), cancer (Benoot and Bilsen 2016;), and bereavement (Butler et al. 2017), in addition to those working on topics relating to violence and abuse (Nikischer 2019). These topics have risk of serious harm or death in common and, as a recent revision of the Diagnostic and Statistical Manual of Mental Disorders (DSM–5) also indicates, extend the populations seen as potentially vulnerable to developing ST to include anyone with indirect exposure to aversive details of events where a person has been ‘exposed to: death, threatened death, actual or threatened serious injury, or actual or threatened sexual violence’ (APA 2013). Some may consider this definition too broad, as it includes the potential for anyone to be defined as vulnerable and therefore any researcher engaging with humans to be traumatised as a result. However, we would argue that there are sensitive areas of research where this risk of negative impacts on researchers is judged to be high, where additional support measures ought to be considered. These additional measures might include Ethics Committees reviewing the emotional safety of research in protocols (as suggested by McKenzie et al. 2016) or clinical supervision as we suggest in this article.

For GBV researchers, there may be potential compounding factors relating either to exposure to traumatic information, or to the people who have experienced the trauma. The first is that the trauma has resulted from the actions of another person, most often someone close to the victim-survivor. This person has intentionally chosen to inflict pain, harm, violence, and/or abuse, and Herman describes how this brings those studying in this field face-to-face with ‘the capacity for evil in human nature’ (Herman 2015). This invariably challenges the way we see the world and humanity, and can potentially impact on both our sense of safety, and the way we might relate to others (Biruski et al. 2014). The second dynamic is that because stigma, embarrassment, shame and guilt often exist around the experiences of abuse, participants may not have communicated their experiences previously. Klein (2012) describes the possibility of participants making their first-ever disclosure of sexual violence in response to questioning by an interviewer. Consequently, when given an opportunity to speak in a safe environment, with someone independent of their situation who is empathetic, people may share material which is raw and unprocessed, finding a release of expression, and pour out detailed descriptions of events and incidents, also allowing themselves to feel, at depth, their emotions (Ellsberg and Heise 2005). This is often experienced as an empowering, cathartic and purging encounter for participants, (Smith 2000; Moch and Cameron 2000; Hutchinson et al. 1994) but can leave a researcher reeling from the deluge, and feeling besieged and unsteadied (Klein 2012). A third factor which might amplify experiences of ST is personal experience. It is not uncommon for people who have experienced GBV in their own life to gravitate towards an active role in this topic of research, including undertaking related research. This ‘insider view’ is incredibly valuable and may, through enhanced empathy with participants, lead to more in-depth and nuanced research findings. However, for researcher protection and self-care, the possibility that exposure to other people’s experiences may trigger memories relating to personal trauma, or may increase cumulative effects of exposure, needs to be considered (Ellsberg and Heise 2002).

In addition to the topic area, the types of research conducted and the models used may unintentionally amplify secondary distress for researchers. Qualitative methods are frequently used, which not only bring researchers face-to-face with people who have experienced trauma, but also require researchers to remain immersed in the data over lengthy periods, through the iterative processes of data collection, transcription, coding, analysis and paper writing. In addition, research in this field tends to attempt to conduct *with* people rather than *on* people, and to incorporate feminist ideals around reducing power imbalances between researcher and participant (Legard et al. 2012). There are bodies of work relating to Participatory Action Research (Burke et al. 2017) and Indigenous Knowledge Research (Marzano 2009) which would critique whether such approaches achieve genuine collaboration, and these criticisms are acknowledged. However, attempting to conduct research in this way requires researchers to be fully present and fully congruent, with a shared sense of humanity with participants, honouring emotional connectedness with people (Williamson et al, 2019a). When done successfully, this certainly enhances the research, but it can also increase the risk of ST due to, what Figley describes as, the ‘cost of caring’ for others in emotional pain (Figley 1982).

The aim of this paper is to explore reflexively the impact of research on the lives of GBV researchers, and the ways in which they guard against ST. We draw on the experiences of a team of researchers who recently completed a large study looking at GBV, although we believe that this paper can contribute to wider discussions about the wellbeing of researchers working in a range of subjects.

## Methodology

This paper is based on the experiences of a research team working on a large-scale project exploring GBV. The wider project involved literature reviews, analysing police and women’s support services’ data, and conducting and analysing interviews with 251 victims/survivors of GBV. The interviewing team consisted of ten researchers, nine women and one male. Relevant to some of the themes that emerged, three of the ten researchers were based off-site, two working from home, and the third at another institution.

Further information about the project, including processes of recruitment, sampling, and data collection can be found elsewhere (Williamson et al. 2019b). This paper, however, is concerned with reflecting on the ways in which the team addressed emotional safety and well-being within the project, and the mechanisms which were put in place to support researchers and to recognise and address problems should they arise.

Ethical approval for the project itself was obtained from the University of Bristol, Faculty of Social Science and Law Research Ethics Committee. A further amendment to the ethics document was requested following the data collection, to ensure that this specific paper could be written without causing distress to the researchers in the team. The lead researcher for the qualitative data collection was aware that a number of the research team, herself included, had been affected at various points with the emotional impact of the interviewing and data collection process. The team discussed this both at length, and on a regular basis. When bringing together some of these reflections and experiences of the team, we did not want the team to have concerns that any confidences would be broken, or personal information used within the paper, unless this was something which individuals explicitly wanted to do. As such, the amendment to the REC asked for permission to write an email to team members to reassure them about how the paper would be written. Permission was granted by the REC and an email sent to all researchers who took part in the qualitative aspects of the project (*N* = 10). All researchers were asked, if they wished to contribute and take part, to contact the co-lead to arrange a discussion of the issues and to talk about ways in which the research had impacted on them. Consent was discussed with researchers at this point in terms of how the information they shared would be used in the paper and that they would have the right to see how extracts from them had been included. Five researchers made contact with the co-lead and discussed, either in person or via email, the issues they wished to raise about the impact of the project.

As the team was small and known to one another, no identifications are offered linked to the extracts used in the findings below. This includes whether the researcher was based on or off site, unless they explicitly mentioned it in their contribution. This was to help anonymise the identification of individual contributors. The themes below and points of reflection are based on these conversations and contributions. Contributions from all researchers who responded are included in this paper.

In terms of analysis, written comments and the text of oral conversations were thematically coded and included within the analysis by the lead researcher. All extracts and the themes were shared with the relevant individual researchers who all agreed with the analysis of the data they had provided. All of the themes which researchers raised are included in this paper. It is important to note that some researchers might have experienced different impacts, or none, from the work but chose not to discuss these for the purposes of this paper. Whilst this could be viewed as a bias in the data being reflected on, it also highlights the problem being discussed. If researchers do not want to discuss the impact of this type of work with their managers, or if we assume that problems don’t exist (as is currently the case), then opportunities to provide support are reduced.

Finally, in terms of method, we recognise and share principles with the autoethnographic approach used by Nikischer (2019) when reflecting on similar issues. The ‘data’ provided and reported in this paper comes from individual researcher reflections and as such is autoethnographic. It has however been analysed and presented by a third party in order to bring together different perspectives to add depth and breadth to the perspectives being offered as well as offering a level of anonymity for those wishing to contribute.

## Supporting Emotional Safety and Well-Being

Before conducting any of the qualitative and quantitative research, we put in place a standard researcher safety protocol, submitted to the Research Ethics Committee, which specifically mentioned emotional safety.

This protocol^2^ recognises that emotional wellbeing is important, and additionally flags this as something which the research team is concerned about. The protocol could be construed as directive, as it requires researchers to be aware of the potential emotional impact of the research process and act on it. It was implemented during the project by team members checking-in with each other after interviews, particularly if they were off-site conducting fieldwork. It was also implemented by having an open-door policy for staff if they needed to talk something through, and by the team as a whole, making themselves visible and available to each other. More formally, we also included emotional well-being on the agenda for every team meeting, and used this as an opportunity to raise and share issues across the team. Any key points and actions which emerged from these discussions were noted.

We also note that transcribers too can be impacted by listening too this kind of interview (Gregory et al. 1997) and the lead researcher in this phase made regular contact with the transcriber to check-in with them and offer support if it were needed.

In practice, team members had a variety of ways of dealing with the emotional impacts of the fieldwork (both interviews and analysis of police data), with some favouring 1:1 and others favouring discussion in team meetings.

*Having this protocol in place was definitely appreciated and made me feel that even though I am <off-site>, the team did care about my emotional well-being– I wasn’t always proactive at calling in every time I had completed an interview – mostly because after an intensive interview I generally took time to myself to calm down, to reflect etc plus taking the time to complete the participant overview spreadsheet immediately after an interview provided some down time so you didn’t leave your desk still thinking about that person and their story. I did find that de-briefing 1:1 was better than within the team meetings.*

## Triggers to ST or Negative Impacts

As outlined earlier, ST has been defined by the American Counselling Association as the ‘emotional residue of exposure’ (Ursano et al. 1999). The content which might act as a trigger to researchers was not only unique to individuals, but were also unable to be predicted in advance. Just as the potential emotional risk to participants is unknown in terms of impact, we did not know what the impact on researchers might be. As outlined in the introduction however, the term secondary trauma is not ideal but it is important to recognise where this paper sits in relation to the wider literature on the impacts of working with trauma as the focus of ones work.

Team members recalled a number of instances where the research had a profound impact, leading to their own disclosure of abuse and/or frustration and anger at the lack of solutions/resolution available for participants:

*For me, within this research, it was a participant talking about the verdict of the inquest into the Hillsborough Disaster*^3^*that had the most impact. It had nothing directly to do with her multiple experiences of rape, but this participants’ hope, following that verdict that justice might be possible, and my overwhelming fear about what would happen if that didn’t happen for her. The possibility of justice was something that stayed with me long after the interview and it led me to decide to report to the police my own historic case of child sexual abuse.*

*I can recall two interviews that had a big emotional impact on me. The first was a woman who had suffered domestic abuse, but had not wanted to deny her young daughter contact with her father – she agreed to joint custody despite what she had gone through thinking it was the best interests of her child- however she has seen changes in her daughter and was totally regretting allowing the daughter to live with her father as she suspected that he was being emotionally abusive to her now too – she was in tears during the interview because she felt so helpless to protect her child and I felt her pain, it was very emotional and I felt very angry, frustrated and helpless afterwards.*

*The second was a woman who had suffered prolonged sexual abuse by partner and family – she didn’t feel able to do an interview in the end but because she had my [work] mobile phone number (which I used to text her) she texted me a couple of times (at 10pm and 6am) saying how she couldn’t sleep and how awful she felt. I had limited text conversation to try and make her feel better and advise her on getting help and also contacted her support worker as soon as I was able to let her know. Not a good position to be in really but to be expected as we were including talking to people who had not necessarily talked through their issues with specialists/professionals first.*

In cases like these, even though the involvement with the participant is not extensive, it can leave the researcher with that feeling of ‘unfinished business’ or feelings of on-going concern and worry for the fate of the person (Burr 1995). It also raises feelings of having an ethical responsibility as a researcher of vulnerable people (Stalker 1998).

*All of the interviews were difficult because you feel empathy, anger, a sense of injustice and helplessness when someone is describing something so terrible happening to them. On top of this, having worked on the issue of sexual exploitation for several years, one particular trigger for me was when participants described this form of abuse. Specifically, a couple of women who had experienced brutal (physical and psychological) domestic violence described how their partners also advertised them online and forced them to have sex with other men – one of the women was heavily pregnant during some of this. This was extremely upsetting because, in addition to these women already experiencing sexual violence from their abusive partners, they were also being raped by several other perpetrators; it also makes you angry that these men would come and pay for sexual access to women who were clearly being pimped by someone, were obviously vulnerable and were not consenting.*

These testimonies from the team highlight a number of factors. They illustrate the emotional impact of conducting research and how this can be linked to injustice, a lack of being able to make some things better, and feeling that researchers might have made things worse. Researchers, unlike front line support staff, are not offering or providing a service, although they are trained to signpost to appropriate support.^4^ This unique role as a researcher can be difficult when it results in researchers feeling unable to intervene or make things better.

It became apparent that triggers could be linked not only to actual interviews, but also to secondary data collection; in this case, detailed police files about rape and domestic violence which were reviewed as part of the study. The trauma here was described as being caused, in part, by not having a link to the person under discussion in the reviewed documents, and also as a result of very factual and detailed ways in which the information about horrific events was conveyed:

Reading through police case files could be just as depressing and upsetting in some of the worst cases and especially the cases involving child victims of rape and family abuse. The police files /child sex abuse cases were particularly hard because of the language and detail of information I was reading – very matter of fact descriptions of the physical sexual acts/ abuse (which I didn’t hear generally during the interviews with victims/survivors). There was also a time when I was collecting data on a DV case and there was a warning attached to the victim’s file which said *DEAD* so I had read all about her history of domestic violence, family abuse, drug and alcohol abuse and then found out that she had actually been found dead 2 weeks after the latest incident and her partner had [previously] been arrested on suspicion of her murder but no further action had been taken (when you could see the pattern of abuse she had suffered and was obviously extremely vulnerable) - that made me gasp out loud in the open plan (and quiet) office I was in (embarrassing) and made me incredibly sad. I cried on my drive home that day.

Clearly, examining this type of data for analysis is ‘part of the job’ of a researcher, and this will be examined shortly, but in the course of conducting this type of work, even if desk based, there will be times when researchers may need additional support to process the emotions they experience from dealing with such information and in order to prevent burnout, as described in the background section. As also mentioned, they may not feel comfortable raising this with their academic line manager, for fear of being perceived as weak and/or unable to do their job. These types of emotional impacts are commonly discussed in counselling literatures (Sanderson 2013) and also on the margins of discussions around reflexive qualitative methodological approaches (Etherington 2009).

Including trauma experienced when looking through case notes may appear questionable to some readers. There is an inherent contradiction in the statement made above that researchers can be impacted negatively when they are close to the ‘research subject’, and also impacted when not close, as in the case of case file analysis. Referring to the impact on researchers reviewing case files of sexual violence, research in the 1980s and 1990s also found that researchers using unobtrusive methods experience similar physical and emotional impacts to those who use in-depth interviews (Milling-Kinard 1996, in Liamputtong 2007).

There were occasional notes of optimism too, where team members described feeling hopeful following exposure to data that indicated professionals’ efforts on behalf of victim-survivors:

*On a more positive note about working with the police files it did also give me a bit of hope about police practice in this area - after reading the comments and efforts employed by individual officers and getting the impression of how far some officers go to investigate the incidents/allegations and how overall victims were being believed and there was inter-agency working and frustration on the part of the police regarding cases not proceeding to prosecution for whatever reason…*

Whilst not explicitly mentioned by the team for this study, we also recognise that reading difficult material within the wider literature can have emotional impact for some people. This means it is important not to assume that desk-based work will be automatically less emotionally traumatic. This also means that we need to recognise that individuals employed to transcribe or manage this type of data need also to be considered. We regularly checked in with the individuals contracted to transcribe our interviews, something which the transcriber commented on and was grateful for, although they never felt the need to explore with us further.

## The Cumulative Impact

Within caring professions, there is often an assumption that more experienced staff have learnt ways to manage stress and are therefore better able to deal with the impacts of emotional work (Ellsberg and Heise 2005). Talking to one of the researchers, this can be a problematic supposition. For some people, that greater knowledge and experience adds to a “crystalline memory” where a trigger, whether a research-related interview, or something in the wider context of the person’s life, such as a book or a film, can pull out those memories and traumas, and actually compound the impact of a trigger point.

*You think it would get easier over the years, but it doesn’t. The fact that we keep having to have these conversations is in itself depressing on top of the nature of the issues we are dealing with.*

This illustrates how, no matter how long we may have been in the field, sometimes as researchers we still underestimate our own emotional well-being in undertaking research with vulnerable people (Liamputtong 2007:87).

The longer-term impact of dealing with these narratives, without clinical supervision as a matter of course, is that some people reach a point where they can no longer immerse themselves in this type of information, choosing to move away from the work, or remaining, but making a choice to work on the issue of GBV within a broader political context. We might perhaps suppose that front line work is even more impactive than research, in terms of ST, however, as this team member described, it has the advantage of feeling more ‘effective’:

*For me it’s the other way around – working within the broader political context … and then becoming immersed in the actual narratives and experiences made me feel like nothing much has changed in terms of the extent that abuse still exists on a massive scale and is allowed to continue – despite decision makers becoming more aware through improved knowledge/research over the last 20 years this doesn’t always translate to the victim/survivor. I know cultural change takes time and most of the time it feels like we are facing an uphill battle in tackling violence against women and girls but for me this project has made me feel like I might be more effective or immediate in helping v/s if I was working more closely/practically.*

## Competing Demands

With any research there are time and resource pressures which managers need to take on board. This can create conflict if trying to balance the demands of the project and the emotional needs of the team. For example, if there are time pressures to complete a certain number of interviews within a certain amount of time, and the researcher requires additional time to process the emotional impacts of the research, these two factors can come into conflict. At a recent conference where these issues were discussed (Williamson and Gregory 2017) a member of the audience, referred to this as an expectation to just “suck it up”, or in other words, for researchers to just get on with the job. This potential conflict between selfcare and getting the research done is important to address. Within the current project, there were very clear targets and timeframes. We were required to conduct at least 240 interviews^5^ over a nine-month period, and extract and collate police data relating to about 900 domestic violence and rape cases over 12 months. By raising the issue of emotional impacts at the outset, we were able to address the potential conflicts directly, and discuss possibilities for the boundaries of support. The question arises, therefore, about what is a legitimate expectation of staff employed to work in what is clearly an emotionally difficult area? On the one hand, it is someone’s job to conduct the work. On the other hand, there are employer responsibilities to take care of employees. Getting the balance right is not always straight forward. For us, having a supportive team made a big difference. We could afford for someone to take a couple of days away from fieldwork, because others could pick up that work until the individual had had time to deal with the impacts and return to fieldwork. Where required, we could shift the types of interviews people were conducting. This was only possible because the team worked together in a reciprocal way to support each other, so that they too could benefit from that support when needed. This would undoubtedly be more difficult in a smaller team and impossible where individuals are working alone. In those circumstances, individuals would feel a greater sense of pressure to get on with the job, irrespective of how they were feeling.

Ultimately, there may be occasions when the experience of conducting this type of research leads to a realisation that this might not be the right job for an individual. This did not happen in this particular project, but has been experienced on other projects. This may be a difficult thing to manage, but is an important consideration when the emotional well-being of an individual researcher is at stake. If someone is not able to attain relatively healthy coping mechanisms, then continuing in this field of work is likely to be traumatic for them, their managers, and their colleagues, in the long-term. Having a policy that recognises emotional well-being, supporting colleagues to do that work, comes alongside this acknowledgement that research is not to be undertaken whatever the cost to researchers. This type of conversation is likely more difficult if taking place between a researcher and line manager. It is one of the reasons why independent clinical supervision may be more appropriate in this type of research which is discussed in the conclusion in terms of policy implications.

## General Coping Strategies

The issue of coping strategies has been raised in previous sections. These can take many different forms and the list below comprises strategies used across the team. There is a wider recognition (Holahan and Moos 1987) that some of these are healthy coping strategies and others, particularly if not in moderation, are less healthy strategies. We make no judgment on this, but recognise that they are approaches people might use in the short-term, long-term, or both, in order to manage the impacts they experienced. Some researchers have also talked about avoiding certain topics on TV, radio and in books and magazines (Brown 2017; McKenzie et al. 2016).

Comfort Eating and drinking alcohol:

*Definitely mindfulness, meditation, and running (not at the same time!). Spending time with family. Counting my blessings. Also wine, chocolate and binge TV watching.*

*I found that I drank more when I was doing interviews. Something I recognise from previous research. It is a way, unhealthy admittedly, of switching off and shutting down some of the emotions that come with carrying other people’s, and my own, trauma.*

Distractions:

Members of the team also talked about forms of distraction, such as reading trashy (simple, shallow) magazines, watching television and reading fiction, or of sharing the burden by talking to friends or colleagues.

*I particularly like murder mysteries which might seem odd. But it makes a change when the baddy gets it!*

*I found it helpful to talk in general terms about the interviews to friends and my partner, e.g. ‘I spoke today to yet another woman who has been raped multiple times in her life’. However, due to confidentiality it is (rightfully) very limited what we can say.*

*I found it very helpful to talk to colleagues. We could vent our frustration, anger and other emotions at yet another instance of someone (mainly women) experiencing violence and abuse. I found almost no difference in terms of talking over the phone or in person [with colleagues]– both were helpful.*

Exercise:

Some described the benefits of exercise, particularly if it was outdoors.

*Taking a walk/getting fresh air. I would find myself getting very caught up in some interviews and in somewhat of a depressed/anxious daze afterwards. In these cases I found that sometimes the best course of action was to stop doing work related to the interview(s) (e.g. entering data from it) and to go outside for a walk. The fresh air, change of scenery and quiet of the park I would walk in always helped.*

Counselling/Therapy:

Others mentioned the need for therapeutic counselling, acknowledging that it might be helpful, but that it is rarely available for researchers:

*Counsellors get clinical supervision to help them deal with the emotions they engage with within the therapeutic relationship. We, as researchers, don’t get that yet we are dealing with many of the same issues.*

Standing Still:

Some described feeling like a “tsunami of emotion” was washing over them, and that at times the only thing to do was to “stand still and let it pass”. This means recognising that there are limits to the amount of emotional impacts people can absorb, and that having the space to stop and reflect is crucial. The processes we put in place for the team were designed to allow space for this reflection to take place. This can only happen, however, if researchers feel able and safe within the team, or with their line manager, or have other avenues to raise concerns when they arise.

The examples above illustrate how this team used a wide range of strategies with varying degrees of success. The team also raise the issue of ‘clinical supervision’, something which is routine in a therapeutic context, but not very common in research. This is something with Liamputtong (2007) raises in relation to providing access to a professional confidante and formal supervision, which includes both academic and therapeutic supervision.

## Rewards

This paper has so far looked at the emotional impacts of conducting research in the field of GBV, and the potential emotional safety issues which arise. There are also (Edwards 1993; Abrahams 2017) rewards which come from sharing someone’s story and ‘actively listening’.

The broader rewards for researchers in this project were about the potential of the research to bring about change:

*As well as the negative impacts of the interviews I mentioned above, those same interviews gave me a real sense that this research could make a difference to real people and real lives. That was a privilege to be a part of, and although difficult, made coming into work worthwhile.*

And this was apparent on a person-to-person level too, where victim-survivors expressed the ‘usefulness’ to them personally of being involved in the research and having had the opportunity to share their experiences

*Having participants thank me for listening, and thank our team for doing this research, was always massively rewarding. Even if I felt helpless regarding their experience of violence/abuse, I felt somewhat better if they expressed that the interview/research was useful.*

An additional reward was remarked upon where the research and the experiences of individual participants, created an opportunity for the researcher to perceive their own life in a different, perhaps more favourable light:

*It was difficult to do the interviews (and I have a lot of experience doing similar interviews) but it was also very rewarding. And I guess it taught me something about myself, about the things that matter to me and how lucky I am compared to some of the women I spoke to, but how much we share as well.*

Finally, of the 251 victims/survivors who we interviewed within the main project, over 30% had, subsequent to the abuse they had experienced, become involved in politics or organisations as a way to respond to the abuse they had experienced and in their search for justice (Williamson et al. 2019a). This reminds us that one of the rewards of conducting research in this field is that, for many of us, we may do this kind of research because we believe that we can contribute to social change and social justice. As with some of the participants who went on to work in this field and were seeking justice for others where they had not got justice for themselves, we too may be engaged in that process of seeking justice not only for others, but for ourselves. In this regard we need to recognise that, where this is the case, it brings both resilience and risks to the researcher with experiential knowledge of GBV.

## Conclusion

Ultimately, many of us continue to carry out research in this field knowing that the work may have a negative impact. As such, we negotiate a balance between rewards and potentially traumatic impacts. We employ a range of healthy and unhealthy coping strategies to deal with the immediate and long term affects, but do so knowing that we are choosing to continue to work in this area, for many, because we want to make social change for those that have and/or may ourselves have experienced or witnessed abuse.

Others, such as McKenzie et al. (2016) have suggested that the review of emotional safety through Research Ethics Committees may be an appropriate way forward, but this is something that already routinely happens at the institution where this research took place, and a comprehensive researcher safety protocol was used. This doesn’t however address the issue of what additional support might be appropriate for researchers working on trauma related topics.

The project on which this paper is based succeeded, in terms of meeting the original target of interviews, and giving victim/survivors a voice, because it was conducted by a team of experienced individuals who had a range of options made available to them to help address the impacts of working in this area. This was identified as more difficult for those staff working remotely, as they have been located outside the main site. This mainly affected the ability to have informal check-ins with others in the team, although they did still feel supported by the fact that support was there if needed. There were no differences in the coping strategies used by those working off the main site. However, not all researchers have the opportunity to work in a team. Many work in isolation, outside of a team context, and unless they are provided with ‘clinical’ or in-depth supervision, which is very rare, are at risk of experiencing some level of trauma themselves when listening too, and engaging with, data of this nature (Nikischer 2019). In most cases academic funders and employers don’t explicitly recognise clinical supervision as an essential part of this kind of work, so it continues to be seen as something that is ‘just part of the job’ (Dickson-Swift et al. 2008, 2009). As outlined above, counsellors who hear very similar stories routinely get clinical supervision to address the emotional impacts. We also recognise that balancing the requirements of getting the job done, and the emotional impact of such research is not something that can always be discussed within the researcher/line manager relationship. As such, a proactive clinical supervision arrangement may provide better safeguards than a reactive approach to addressing the needs of researchers in distressing sensitive subjects. This might take the form of a pre-fieldwork session to identify potential triggers and coping strategies and bi-monthly check – ins so that arising issues can be identified and discussed. This conclusion echoes those of Dickson-Swift et al. (2008, 2009) who recommended that: “professional supervision, policy development, and minimum training standards for researchers are provided” (Dickson-Swift et al. 2009).

Undermining the suggestion here about clinical supervision is the issue of cost and funding. We are aware in the current economic context that ring fencing funding for clinical supervision may be seen as a luxury that academic institutions and research funders can ill afford. However, we would question whether funders and employers are doing enough to support researchers in dealing with the emotional impacts of their work and thus avoid the burnout which can result. Losing experienced and highly trained staff is not an insignificant cost to these institutions.

For many of the researchers in this project, the greatest emotional impacts were linked to frustrations related to feeling that socially nothing has changed, sometimes described as hopelessness. Conversely, the rewards related to making a difference by hearing people’s stories and giving them a voice. With this in mind, the importance of public engagement and impact of research cannot be underestimated. Whilst research generally has moved in recent years to recognise the importance of ‘impact’, this is something which has been central to the GBV field from its outset (Lilley (Walker) and Hester 2012).

To conclude, researching GBV is hard, but the negative emotional impacts or ST which researchers may experience can, for many people, be managed through support and understanding. Whilst the work is difficult, it also offers the reward of making a difference, and giving a voice to those who are often marginalised and silenced. For some researchers however, the impact of researching GBV and abuse will be damaging. From a policy context we would like to see funders and institutions recognise the potential benefits of clinical supervision being available for researchers working with sensitive issues, whether interviews or data derived from such populations/participants. There should be additional specific funding so that those applying for research funding, and who need such support, are not penalised financially for including such support within their proposals. We anticipate that this would be as little as 2% of any project grant. We hope to continue such policy discussions with the aim of ensuring that the impacts of research are better recognised, acknowledged, and that different options and safeguards are considered to support researchers working in all areas of research dealing with trauma and/or abuse.

## Footnotes

1 The WHO work is focused on women who are victims of abuse. Listening to others accounts of abuse will be the same.

2 “Emotional wellbeing: Researchers will be aware that this kind of work can be emotionally demanding. Debriefing: 1) Researchers will plan time after each visit where they can debrief about visit and interviews undertaken. This can be done immediately after the visit, by telephone or in person. Where possible, this should happen within 48 h. 2) In regular team meetings, the team will discuss any emotional aspects of the work and address any particular issues which have arisen. Where a member of staff has experienced a difficult situation, s/he will seek support from colleagues. If, after debriefing and further meetings with colleagues, the researcher is still in need of support or if s/he would like to speak to a Counsellor, s/he is free to do this at any point. Work-based counselling services are available by contacting <details provided>.”

3 The Hillsborough Disaster occurred at a football match in 1989. 96 people lost their lives with hundreds injured. Despite repeated calls from the families for an inquest and inquiry, it took until April 2016, at a second Coroner’s inquest, before the true cause of the failings were acknowledged. The Coroner ruled that supporters were unlawfully killed due to grossly negligent failures by police and ambulance services to fulfil their duty of care.

4 This support included information about local support services for relevant types of issues, for example: for sexual abuse, domestic violence, as well as national helplines providing further signposting.

5 The project was concerned with justice, inequalities and gender based violence. In order to ensure that the different permetations of experience were captured in the research we identified a number of rubrics which we would need to fill which would necessitate at least 240 interviews. Further information available <Williamson et al. 2019a>.
